# [Retracted] Recent perspectives of electronic medical record systems (Review)

**DOI:** 10.3892/etm.2026.13126

**Published:** 2026-03-09

**Authors:** Xiao-Ying Zhang, Peiying Zhang

Exp Ther Med 11:2083–2085, 2016; DOI: 10.3892/etm.2016.3233

Following the publication of this article, an interested reader drew to our attention that the flow of the text, and the wording of numerous passages of text within the article, bore striking and unexpected similarities to the wording employed in a PhD thesis by Marlen Stacey Chawani at the University of Oslo, Norway, in 2014 called “Development of Electronic Medical Record Systems for Maternal Health Services in Rural Settings”.

After having conducted an internal investigation, the Editor of *Experimental and Therapeutic Medicine* has determined beyond all reasonable doubt that the review submitted to our Journal shared an unacceptable level of identity with the PhD thesis by Marlen Stacey Chawani; therefore, this review article is to be retracted from the Journal. The Editor of *Experimental and Therapeutic Medicine* deeply regrets that this article was not intercepted by our own checks for plagiarism prior to the publication of the review, and sincerely apologizes to the author of the PhD thesis and the readership for the inconvenience caused. We also thank the interested reader for bringing this matter to our attention. The authors were asked for an explanation to account for these concerns, but the Editorial Office did not receive a reply.

